# 

**Published:** 1909-03-20

**Authors:** 


THE
### GRADUATES' MEDICAL SCHOOLS.

DIARY FOR THE WEEK MARCH 22 TO MARCH 27.

**LONDON THROAT HOSPITAL, Great Portland Street, W.**

**At 5 p.m.**
**March 24,** Dr. G. Cathcart, **Impaired Movements of the Vocal Cords.**

**LONDON SCHOOL OF CLINICAL MEDICINE, Seamen's Hospital, Greenwich, S.E.**

**At 2.15 p.m.**
**March 22,** Sir Dyce Duckworth, **Tedious Recovery from Illness.**
**At 3.15 p.m.**
**March 25,** Sir Wm. Bennett.

**THE HOSPITAL FOR SICK CHILDREN, Great Ormond Street, W.C.**

**At 4 p.m.**
**March 25,** Dr. Thompson, **Selected Cases of Nervous Disease.**

**NATIONAL HOSPITAL FOR THE PARALYSED AND EPILEPTIC, Queen Sq., Bloomsbury, W.C.**

**At 3.30 p.m.**
**March 23,** Mr. L. Paton, **Retro-Bulbar Neuritis.**
**March 26,** Dr. Grainger Stewart, **Disseminated Sclerosis.**

**THE POST-GRADUATE COLLEGE, West London Hospital, Hammersmith, W.**

**At 10 a.m.**
**March 22 and 25,** Surgical Registrar, **Demonstration.**
**March 26,** Medical Registrar, **Demonstration.**
**At 12 noon.**
**March 22,** Dr. Low, **Pathological Demonstration.**
**At 12.15 p.m.**
**March 24 and 27,** Dr. Pritchard, **Practical Medicine.**
**At 5 p.m.**
**March 22,** Dr. Saunders, **Clinical Lecture.**
**March 23,** Dr. Low, **Insects as Carriers of Disease in Tropical Medicine.**
**March 24,** Dr. Beddard, **Medicine—V.**
**March 25,** Mr. Edwards, **Clinical Lecture.**
**At 3 p.m.**
**March 26,** Dr. R. H. Cole, **The Medico-Legal Aspects of Insanity** (at Hanwell Asylum).

**MEDICAL GRADUATES' COLLEGE AND POLYCLINIC, 22 Chenies Street, W.C.**

**At 4 p.m.**
**March 22,** Dr. J. M. H. MacLeod, **Clinique (Skin).**
**At 5.15 p.m.**
**March 22,** Mr. E. Clarke, **The Fundus Oculi** (with lantern slides).
**At 4 p.m.**
**March 23,** Dr. E. Cautley, **Clinique (Medical).**
**At 5.15 p.m.**
**March 23,** Mr. L. Mummery, **The Pathological Causes and Surgical Treatment of some Cases of Severe Chronic Constipation.**
**At 4 p.m.**
**March 24,** Mr. Charles Ryall, **Clinique (Surgery).**
**At 5.15 p.m.**
**March 24,** Mr. L. Mummery, **The Pathological Causes and Surgical Treatment of some Cases of Severe Chronic Constipation.**
**At 4 p.m.**
**March 25,** Sir Jon. Hutchinson, **Clinique (Surgery).**
**At 5·15 p.m.**
**March 25,** Dr. H. Walsham, **The Diagnosis of some Obscure Diseases of the Chest by X-rays.**
**At 4 p.m.**
**March 26,** Dr. St. Clair Thomson, **Clinique (Throat).**

**ST. JOHN'S HOSPITAL FOR DISEASES OF THE SKIN, Leicester Square, W.C.**

**At 6 p.m.**
**March 25,** Mr. Chisholm Williams, F.R.C.S., **Demonstration on Electro-Therapeutic Methods in some Skin Diseases.**

